# The absence of dysferlin induces the expression of functional connexin-based hemichannels in human myotubes

**DOI:** 10.1186/s12860-016-0096-6

**Published:** 2016-05-24

**Authors:** Luis A. Cea, Jorge A. Bevilacqua, Christian Arriagada, Ana María Cárdenas, Anne Bigot, Vincent Mouly, Juan C. Sáez, Pablo Caviedes

**Affiliations:** Program of Anatomy and Developmental Biology, Faculty of Medicine, Institute of Biomedical Sciences, University of Chile, Av. Independencia #1027, Independencia, Santiago, Chile; Departamento de Neurología y Neurocirugía, Hospital Clínico Universidad de Chile, Universidad de Chile, Santiago, Chile; Centro Interdisciplinario de Neurociencias de Valparaíso, Universidad de Valparaíso, Valparaíso, Chile; Center for Research in Myology, Sorbonne Universités, UPMC Univ Paris 06, INSERM UMRS974, CNRS FRE3617, 47 Boulevard de l’hôpital, 75013 Paris, France; Departamento de Fisiología, Facultad de Ciencias Biológicas, Pontificia Universidad Católica de Chile, Santiago, Chile; Programa de Farmacología Molecular y Clínica, Facultad de Medicina, Instituto de Ciencias Biomédicas, Universidad de Chile, Santiago, Chile

**Keywords:** Dysferlinopathy, Membrane permeability, Calcium

## Abstract

**Background:**

Mutations in the gene encoding for dysferlin cause recessive autosomal muscular dystrophies called dysferlinopathies. These mutations induce several alterations in skeletal muscles, including, inflammation, increased membrane permeability and cell death. Despite the fact that the etiology of dysferlinopathies is known, the mechanism that explains the aforementioned alterations is still elusive. Therefore, we have now evaluated the potential involvement of connexin based hemichannels in the pathophysiology of dysferlinopathies.

**Results:**

Human deltoid muscle biopsies of 5 Chilean dysferlinopathy patients exhibited the presence of muscular connexins (Cx40.1, Cx43 and Cx45). The presence of these connexins was also observed in human myotubes derived from immortalized myoblasts derived from other patients with mutated forms of dysferlin. In addition to the aforementioned connexins, these myotubes expressed functional connexin based hemichannels, evaluated by ethidium uptake assays, as opposed to myotubes obtained from a normal human muscle cell line, RCMH. This response was reproduced in a knock-down model of dysferlin, by treating RCMH cell line with small hairpin RNA specific for dysferlin (RCMH-sh Dysferlin). Also, the presence of P2X_7_ receptor and the transient receptor potential channel, TRPV2, another Ca^2+^ permeable channels, was detected in the myotubes expressing mutated dysferlin, and an elevated resting intracellular Ca^2+^ level was found in the latter myotubes, which was in turn reduced to control levels in the presence of the molecule D4, a selective Cx HCs inhibitor.

**Conclusions:**

The data suggests that dysferlin deficiency, caused by mutation or downregulation of dysferlin, promotes the expression of Cx HCs. Then, the *de novo* expression Cx HC causes a dysregulation of intracellular free Ca^2+^ levels, which could underlie muscular damage associated to dysferlin mutations. This mechanism could constitute a potential therapeutical target in dysferlinopathies.

**Electronic supplementary material:**

The online version of this article (doi:10.1186/s12860-016-0096-6) contains supplementary material, which is available to authorized users.

## Background

Dysferlinopathies are muscular dystrophies caused by mutations in dysferlin, a 230 kDa membrane protein, mainly localized in the sarcolemma [1] and the T-tubule system [2, 3]. It is accepted that dysferlin participates in membrane resealing after damage [4, 5]. This protein is weakly expressed in myoblasts (myogenic precursor cells), but highly expressed in adult skeletal muscle [6]. Clinically, dysferlinopathies manifest between the second and third decade of life in previously asymptomatic patients. At onset, most patients refer weakness in the lower extremities, difficulty in running or climbing stairs, sometimes accompanied with pain [7]. Regarding the alterations produced by dysferlin mutations in muscle, prior reports have detected the presence of inflammation [8–10]; disruption of the T-tubule structure, which in turn was ameliorated by reduction of external [Ca^2+^] or blocking of L-type Ca^2+^ channels with diltiazem [3], suggesting that a deregulated entry of external Ca^2+^ may underlie damage in dysferlin null myofibers. In addition, an altered permeability to dyes such as Evans blue has been reported in skeletal muscles from Dysf^-/-^ mice (an animal model of dysferlinopathy) [4], and previous reports have demonstrated that Evans blue crosses the cell membrane through Cx HC [11]. Hence, these results strongly suggest the presence of connexin based hemichannels (Cx HC) in the sarcolemma of myofibers from the animal model. As we previously reported, the *de novo* expression of Cx HCs has been observed in similar pathologies, where they mediate myofiber atrophy induced by denervation [11]. Interestingly, only a mild muscular atrophy was observed after denervation in Cx43 and Cx45 KO mice [11]. Since Cx HC are non-selective channels permeable to ions (e.g. Ca^2+^ and Na^+^) and small compounds, including signaling molecules such as ATP and NAD^+^ and dyes including ethidium (Etd^+^) and Evans blue [12, 13], the altered membrane permeability caused by the Cx HC expression could contribute to the development of the muscular atrophy. Indeed, the *de novo* expression of Cx HCs promotes the increase of oxidative stress in pathological conditions such as muscle denervation [14] and they constitute a mechanism of ATP release in several muscle pathologies [11, 12, 14].

To date there is no effective treatment to arrest or even reduce the symptomatology of the patients affected with dysferlinopathies. Nevertheless, the introduction of a mini-dysferlin in animal models of the disease (Dysf^-/-^ mice) results in the recovery of membrane resealing function. However, the progressive degeneration, ascertained from muscle histology studies, remains unabated [15]. The aforementioned evidence points to the existence of an additional pathological mechanism, triggered by the absence of dysferlin. In the present work we evaluated whether myotubes of patients suffering from dysferlinopathies, as well as in other in vitro models of dysferlin deficiency, express Cx HCs and whether the expression of these types of channels alters the sarcolemma permeability, and increases intracellular free Ca^2+^ in these cells.

## Results

### Human muscles bearing dysferlin mutations express connexins 40.1, 43 and 45

**Fig. 1:**
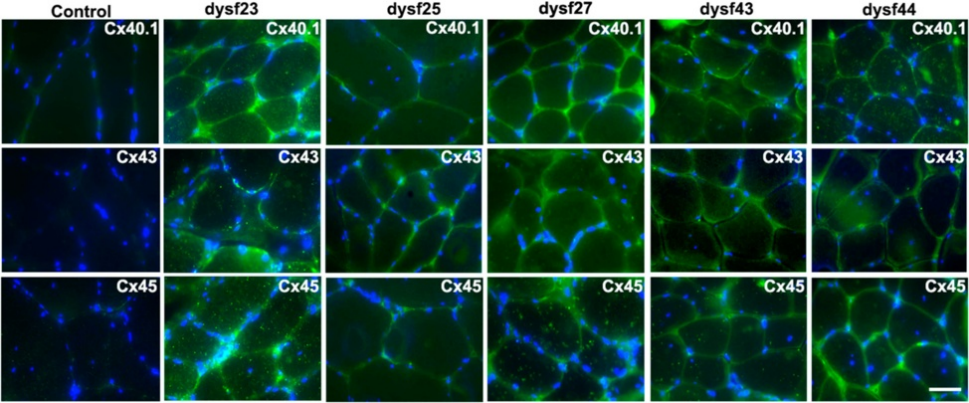
Connexins 40.1, 43 and 45 are present in human biopsies from dysferlinopathy patients. Connexin 40.1, 43 and 45 were detected by immunofluorescence assay using specific antibodies in muscular biopsies obtained from five dysferlinopathy patients at the University of Chile Clinical Hospital, and from a patient that not bear a muscular pathology (control). Cell nuclei were stained with DAPI (blue signal). Scale bar: 50 μm

**Fig. 2:**
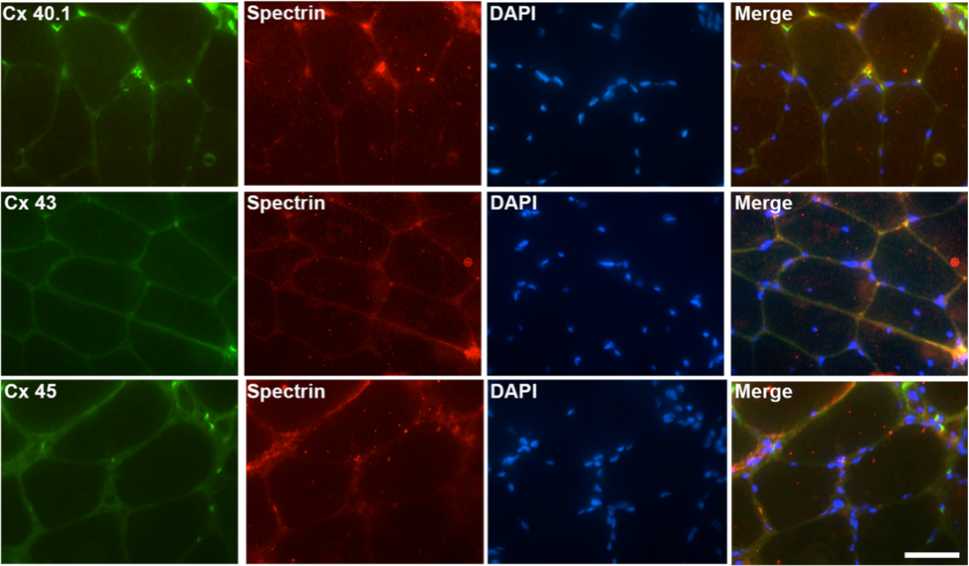
Connexins 40.1, 43 and 45 are distributed in sarcolemma of human muscles biopsies from dysferlinopathy patients. Connexins 40.1, 43 and 45 (green signal) and the sarcolemma protein spectrin (red signal) were detected by immunofluorescence assay using specific antibodies in muscular biopsies obtained from a dysferlinopathy patient (dysf 25). Co-localization of a connexin with spectrin is denoted by the yellow signals. Nuclei were stained with DAPI (blue signal). Scale bar: 50 μm

**Fig. 3:**
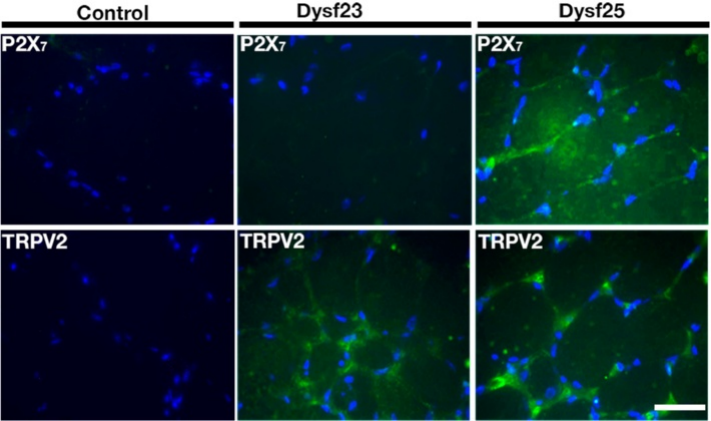
Presence of P2X_7_ receptor and TRPV2 channel in muscular biopsies from dysferlinopathy patients. The purinergic receptor P2X_7_ and the transient receptor potential vanilloid type 2 (TRPV2) channel were detected by immunofluorescence studies using specific antibodies in cross sections of human muscular biopsies from two dysferlinopathy patients and a control patient (patient without a muscular pathology). Nuclei were stained with DAPI (blue signal). Scale bar: 50 μm

We analyzed the presence of connexin proteins by immnunofluorescent microscopy in human muscles biopsies from patients bearing dysferlin mutations (see methods for dysferlin mutations), the absence of dysferlin was confirmed by immunohistochemistry assays (data not shown). As shown in Fig. 1, connexins 40.1, 43 and 45 (green signal, Fig. 1) were detected in biopsies from patients with dysferlinopathy but not in biopsies of control subjects (control). These proteins colocalized with the plasma membrane protein spectrin (Fig. 2) [16], indicating that all three connexins are present in the sarcolemma. Using immunofluoresence, we next evaluated the presence of the purinergic receptor P2X_7_ and the transient receptor potential cation channel subfamily V member 2 (TRPV2), which have been previously associated with muscular atrophy [11]. P2X_7_ receptors were detected in one of the two patients evaluated, whereas TRPV2 was found in the biopsies of both patients (Fig. 3). Conversely, in control patients (patients without a muscular pathology) both receptors were absent (Fig. 3).

**Fig. 4:**
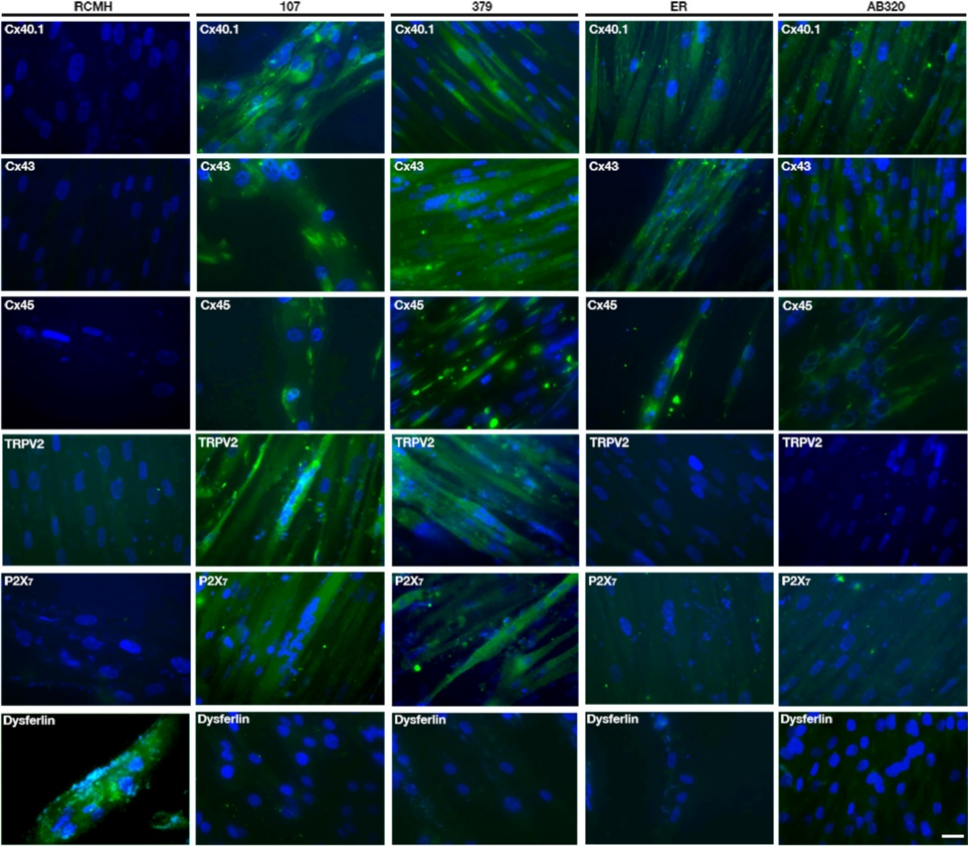
Human dysferlin-mutated myotubes present connexins 40.1, 43 and 45. Human dysferlin-mutated myotubes were obtained by differentiation of dysferlin-mutated myoblast lines named 107, 379, AB320 and ER, each one bearing different dysferlin mutations. Connexins 40.1, 43 and 45 were detected by immunofluorescence studies using specific antibodies. All cell lines showed positive reactivity to Cxs (green signal) compared with control myotubes (RCMH), where Cxs reactivity was not detected. P2X_7_ receptor and TRPV2 channel were also detected by immunofluorescence studies using specific antibodies. Only 107 and 379 myotubes expressed P2X_7_ receptors and TRPV2 channels. Nuclei were stained with DAPI (blue signal). Scale bar: 30 μm. *n* = 4 cell cultures for each cell line

Because Ca^2+^ influx is reportedly increased in human muscles bearing dysferlin mutations [17], we evaluated the presence, in human dysferlin-mutated myotubes (HDMM) (using immunofluorescent miscroscopy), of different Ca^2+^ channels; connexin-based hemichannels, transient potential receptor TRPV2 and P2X_7_, all of which have been previously linked in muscular pathologies [11, 12, 18]. We observed the presence of Cxs 40.1, 43 and 45 in all HDMM, although they have different dysferlin mutations (Fig. 4, green signal), they were absent in normal myotubes (Control, RCMH cells). On the other hand, TRPV2 channels and P2X_7_ receptors were only present in 107 and 379 (dysferlin-mutated cell lines, see methods) derived myotubes (Fig. 4). TRPV2 channels were functional in 379 myotubes, as evidenced by their response to stimulation with 2-aminoethoxydiphenyl borate (2-APB), a selective TRPV2 agonist [19], which induces a sustained increase in Ca^2+^ levels (Additional file 1: Figure S1).

### Absence of dysferlin in control RCMH myotubes mimics the dysferlin mutation effect

**Fig. 5:**
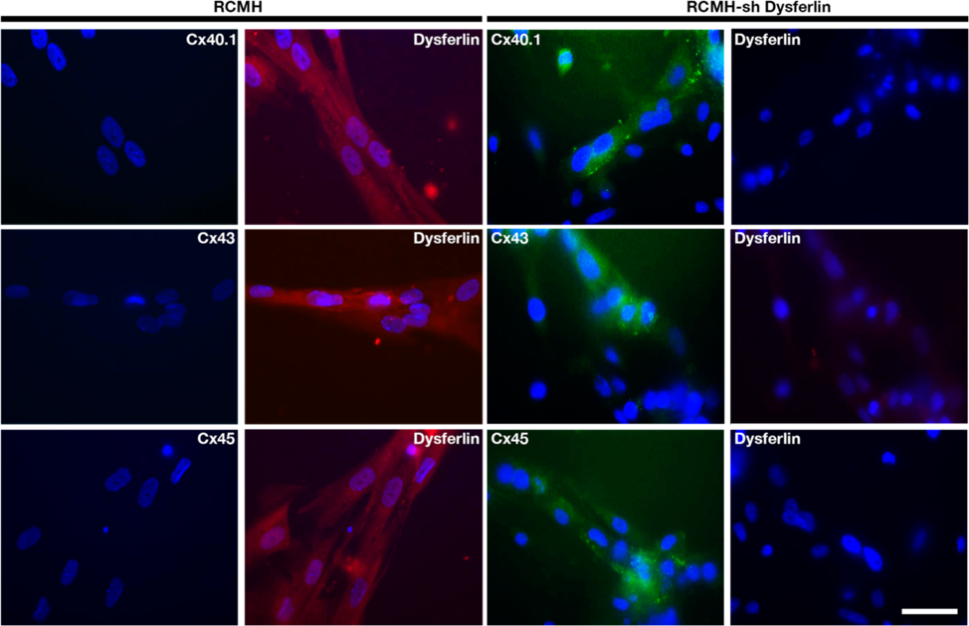
Silencing of dysferlin induces the presence of connexin 40.1, 43 and 45 in human myotubes. Control RCMH myoblasts were transfected with small hairpin RNA to decrease dysferlin expression, to be later differentiated to myotubes by culturing in differentiation medium for 14 days. Once myotubes were obtained, the presence of connexin 40.1, 43, 45 (green signal) and dysferlin (red signal) was analyzed by immunofluorescence assay using specific antibodies and compared with that of control RCMH myotubes (without transfection). The three connexins, but not dysferlin, were detected in RCMH-sh Dysferlin myotubes, whereas control RCMH myotubes present dysferlin (red signal) but not Cxs. The nuclei were stained with DAPI (blue signal). Scale bar: 50 μm. *n* = 4 cell cultures for each cell line

To demonstrate that the absence of dysferlin, regardless of the type of mutation, can induce the expression of connexins in normal human myotubes, we knocked-down expression of dysferlin in RCMH cells by transfecting a small hairpin RNA specific for dysferlin, and transfected cells were subsequently differentiated to myotubes (RCMH-sh Dysf). In the absence of dysferlin, RCMH cells express connexins 40.1, 43 and 45 (green signal), which were in control myotubes (RCMH) that express dysferlin (red signal, Fig. 5).

### Human dysferlin-mutated myotubes express functional connexin based hemichannels

**Fig. 6:**
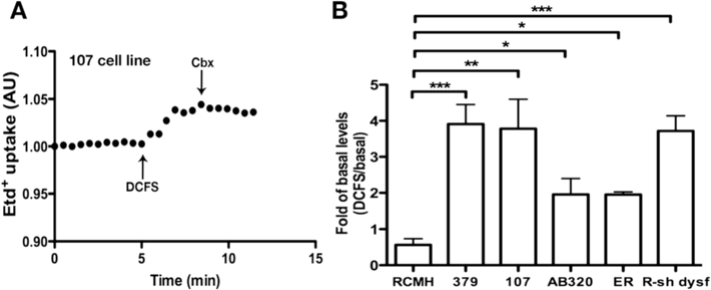
Absence of dysferlin promotes elevated connexin-based hemichannel activity in human myotubes. Human dysferlin-mutated myotubes and control myotubes transfected with small hairpin RNA against Dysferlin (R-sh dysf) were used to evaluate connexin hemichannel activity using the ethidium (Etd^+^) uptake assay. Briefly, **a** Representative curve of Etd^+^ uptake in myotubes differentiated from 107 myoblast cell line. Notice the enhanced uptake after incubation in a divalent cation free solution (DCFS), condition that induces opening of Cx HCs. The signals were inhibited by 50 μM carbenoxolone, a Cx HC blocker. **b** Graph showing the fold variation between the DCFS-induced slope versus basal slope (Fold of basal). * *P* < 0.05; **P < 0.01; ***P < 0.001. *n* = 4 cell cultures for each cell line

Because Cx proteins are present in dysferlin-mutated myotubes (Fig. 4), we analyzed whether these proteins were indeed forming functional hemichannels (Cx HCs) in these cells. Hemichannel activity was evaluated by ethidium (Etd^+^) uptake assays [20], which revealed that HDMM demonstrated elevated Etd^+^ uptake compared with control myotubes (Fig. 6). We detected a 4-fold increment in the 107 and 379 cell lines, compared with RCMH, whereas AB320 and ER (human dysferlin-mutated cell lines, see methods) showed an increment of 2-fold compared to RCMH. Etd^+^ uptake was successfully inhibited with external application of 50 μM carbenoxolone (Fig. 5a), a Cx HC inhibitor [21]. In addition, we observed that different dysferlin mutations exhibit different Cx HC activity. Indeed, cell lines 107 and 379 presented the highest Cx HC activity (Fig. 6b). In addition, RCMH myotubes where dysferlin was silenced via specific small hairpin RNA resulted in the expression of functional Cx HCs to levels comparable to 107 and 379 myotubes (Fig. 6b).

### Inhibition of connexin-based hemichannels reduces elevated intracellular basal Ca^2+^ signals

**Fig. 7:**
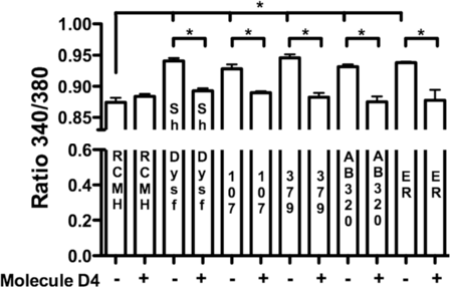
Elevated intracellular basal Ca^2+^ signal in dysferlin-mutated myotubes is reduced by Cx HC inhibitor. Four different dysferlin-mutated myotubes (called 107, 379, AB320 and ER) were used, which were differentiated to myotubes with differentiation medium (DMEM/F12 supplemented with 5 % horse serum plus insulin 10 ug/ml). The Ca^2+^ signal was evaluated using the FURA-2 AM dye in a fluorescence microscope. Signals were compared to those obtained in control myotubes (RCMH). Molecule D4 (D4, 100 nM), a selective connexin hemichannels (Cx HC) inhibitor, was used. During the last 7 days (D4 was added daily during 7 days) before the Ca^2+^ measurement was performed, D4 was applied to prevent the increase of intracellular basal Ca^2+^ signal in human dysferlin mutated myotubes. The data represent as mean ± SEM. ANOVA test with Dunn post test. *P < 0.05. *n* = 4 cell cultures for each cell line

Considering that Ca^2+^ influx is reportedly increased in human muscles bearing dysferlin mutations [17], and that human dysferlin-mutated myotubes express functional Cx HCs (Fig. 6), which in turn are non-selective permeable Ca^2+^channels [22], we analyzed basal cytosolic Ca^2+^ levels in the dysferlin-deficient and control lines using FURA 2-AM assays. Dysferlin-mutated myotubes showed significantly elevated Ca^2+^ levels compared to control myotubes (Fig. 7), a finding consistent with the increased Cx HC activity observed in the four cell lines evaluated herein. Since Cx HCs were present and the basal intracellular Ca^2+^ level was elevated in dysferlin-mutated myotubes, we then investigated whether the inhibition of Cx HCs could prevent this phenomenon. Therefore, human dysferlin-mutated myotubes were treated daily for 7 days with 100 nM D4, a selective Cx HC blocker that does not inhibit gap junction channels, thus allowing the fusion of myoblasts to form myotubes where gap junction channels are relevant [23]. Incubation with D4 significantly reverted the intracellular basal Ca^2+^ signals to values comparable to those of control cells (Fig. 7), suggesting that these channels are responsible of this response.

## Discussion

In the present report, we have demonstrated that muscles from patients diagnosed with dysferlinopathy express Cxs 40.1, 43 and 45 at the plasma membrane. Additionally, four immortalized muscle cell lines generated from muscle samples of dysferlinopathy patients also expressed these Cxs, which in turn, formed functional Cx-based hemichannels. This phenomenon was also induced in control myotubes (RCMH, which normally do not express Cxs), after decreasing dysferlin expression through the transfection (in myoblast stage) of a specific small hairpin RNA against dysferlin. Moreover, human dysferlin-mutated 107 and 379 myotubes expressed two additionals Ca^2+^ permeable channels, the TRPV2 channel and P2X_7_ receptor. These myotubes also exhibited greater Cx HC activity, and such an elevated Cx HC activity could explain the presence of these channels in the myotubes, which in turn could also contribute to the elevated intracellular Ca^2+^ signal previously reported in dysferlin KO skeletal muscle fibers [3] and confirmed here in our model. Anyway, it seems that the presence of Cx HCs is sufficient to explain the elevated basal intracellular Ca^2+^ levels in dysferlin-deficient myocytes, especially if we consider that the specific inhibition of these channels with a selective blocker (molecule D4), was enough to reduce the elevated basal Ca^2+^ concentration to levels similar to those of control cells. The mechanisms regulating Ca^2+^ in myotubes, such as the Na^+^/Ca^2+^ exchanger, Ca^2+^ binding proteins and most importantly the sarcoplasmic/endoplasmic reticulum Ca^2+^ ATPases (SERCA), are extremely efficient in controlling the cytosolic levels of this cation [24]. Even so, integration of Cx hemichannels into the plasma membrane increased the basal Ca^2+^ concentration by ~8 %, which may be enough to alter cellular homeostasis of myocytes. This increment in Ca^2+^ levels could be enough to alter the cellular homeostasis of the myocytes. Indeed, an altered cytosolic Ca^2+^ level could induce several changes in the myotubes, including an increased proteolytic activity [25], which occurs in muscles from patients bearing dysferlin mutations [26]. The latter could then drive atrophy in affected muscles, and also to cause the degeneration of muscles by apoptosis or necrosis [18]. These findings have been previously observed in Duchenne muscular dystrophy were resting Ca^2+^ levels in human DMD myotubes were increased compared with normal myotubes [27].

Therefore, in addition to the deficit in membrane repair mechanism found in dysferlin-mutated myofibers [4], we propose herein a second pathological mechanism as a consequence of dysferlin mutations, which involves Cx HCs. These non-selective channels could explain several of the reported features in dysferlin-deficient muscles, including the increased membrane permeability [4], higher proteasome activity [26, 28], activation of inflammation signals [8–10] and muscle degeneration [29]. The latter interpretation is supported by prior reports showing the role of Cx HC in altered membrane permeability and atrophy of skeletal muscles induced by denervation [11, 12]. Although the mechanism responsible of the induction of connexins by the absence of dysferlin still remains to be elucidated, the fact that the knock-down of dysferlin induces the *de novo* expression of functional connexin-based hemichannels suggests a direct relation between the absence of dysferlin and the presence of connexin. However, it has been demonstrated that some conditions such as inflammation induces the expression of functional connexin-based hemichannels in skeletal muscles through proinflammatory cytokines and the transcription factor NFκB [11, 12]. Inflammation has been described as a key feature in dysferlin-mutated skeletal muscles [9]. Whether the induction of Cx HC involves an activation of NFκB still needs to be investigated.

## Conclusions

In this study, we have demonstrated the presence of connexins 40.1, 43 and 45 in muscular biopsies of patients with different dysferlin mutations. This was confirmed in myotubes derived from cell lines of human dysferlinopathy patients, as well as in normal myotubes where dysferlin was knocked down. The Cxs expressed formed functional connexin based hemichannels, that are responsible of elevated basal intracellular Ca^2+^ levels in human myotubes. The presence of these channels may represent a novel pathological mechanism in dysferlinopathies, as well as a most attractive therapeutical target, considering their localization at the membrane level.

## Methods

### Reagents

Anti-rabbit or anti-mouse IgG antibodies-conjugated to Cy2 (green) or Cy3 (red) were purchased from Jackson immunoresearch laboratories (West Grove, PA, USA). Ethidium (Etd^+^) bromide was from GIBCO/BRL (*Grand Island*, NY, USA), fluoromount-G was from Electron Microscopy Science (Hatfield, PA, USA), Previously described polyclonal anti-Cx40.1, -43, and -Cx45 antibodies were used [11]. Synthesis and characterization of D4 molecule will be published elsewhere.

### Generation of dysferlin-mutated myoblast cell lines

The protocol used was obtained from Philippi et al. [30]. Briefly, primary myoblasts isolated by protease digestion were obtained from fresh muscle biopsies and expanded at 37 °C in skeletal muscle growth medium (PromoCell, Heidelberg, Germany) supplemented with 10 % FCS (Gibco, Paisely, UK). The cultures were enriched in myoblasts by immuno-magnetic cell sorting using anti-CD56/NCAM antibody coated magnetic beads (Miltenyi Biotech, Bergisch Gladbach, Germany). Purity of the myoblast preparation was verified by staining with an anti-desmin antibody (DAKO) revealing more than 95 % desmin-positive cells.

### Immortalization of primary human myoblasts and their differentiation into myotubes

Primary human dysferlin-deficient myoblast lines were transduced with pBABE retroviral vectors carrying Cdk4 and hTERT. Puromycin and neomycin were used as selection markers, respectively and isolation of individual myogenic clones was carried out as described by Mamchaoui et al. [31]. The immortalized dysferlin-deficient human myoblast lines were cultured in growth medium consisting of 1 vol 199 Medium (Invitrogen, Carlsbad, CA)/4 vol DMEM (Invitrogen) supplemented with 20 % foetal calf serum (Invitrogen), 2.5 ng/ml HGF (Invitrogen), 0.1 μM Dexamethasone (Sigma-Aldrich, St. Louis, MO) and 50 μg/ml Gentamycin (Invitrogen). Differentiation into myotubes was initiated at approximately 90 % confluence by cultivation in differentiation medium (DMEM/F12, 5 % horse serum, insulin 10 μg/ml) for 14 days.

Four different dysferlin-mutated myoblast lines were generated and called 107, 379, AB320 and ER, each one bearing a different dysferlin mutation as follow: **107**, c.855 + 1delG c.895G > A; **379**, c.1448C > A c.107 T > A; **AB320**, c.342-1G > A HTZ c.3516_3517delTT (p.Ser1173X) HTZ; **ER**, G1628R(c.4882G > A)HMZ.

### Intracellular Ca^2+^ Signals

Basal intracellular Ca^2+^ signals were evaluated in myotubes by using the ratiometric dye FURA 2-AM. The myotubes were incubated in Krebs-Ringer buffer (in mM: 145 NaCl, 5 KCl, 3 CaCl_2_, 1 MgCl_2_, 5.6 glucose, 10 HEPES-Na, pH7.4) containing FURA2-AM dye (2 μM) during 55 min at room temperature. Then, the Ca^2+^ signal was evaluated in a Nikon Eclipse *Ti* microscope equipped with epifluorescence illumination, and images were obtained by using a Clara camera (Andor), at 2 wavelength (λ) 340 and 380 nm, calculating the ratio 340 vs 380.

### Membrane permeability to dyes

The uptake of ethidium (Etd^+^) was evaluated by using time lapse measurements. Human myotubes plated onto glass cover slips were washed twice with Krebs-buffer solution. For time lapse measurements myotubes were incubated in Krebs buffer solution containing 5 μM Etd^+^ bromide. Etd^+^ fluorescence was recorded in regions of interest located in nuclei of the myofibers. An epifluorescence Nikon *Ti* microscope was used. Images were captured with a Clara camera (Andor) every 30 s. Image processing was performed off-line with ImageJ software (NIH, Bethesda, USA).

### Immunofluorescence analysis

To detect different proteins in myotubes or cross sections (10 μm) of muscles (fast frozen with iso-methyl-butane cooled in liquid nitrogen) samples were obtained and processed as described previously [11]. Briefly, samples were fixed in paraformaldehyde 4 % by 5 min, then incubated at 4 °C for 12 h with diluted primary anti-Cx40.1 (1:100), anti-Cx43 (1:250), anti-Cx45 (1:250), anti-Panx1 (1:300), anti-TRPV2 (1:100) or anti-P2X_7_ (1:100) antibodies followed by 4 washes with PBS 1X and then, incubated with an appropriate dilution of alexa488-conjugated goat anti-rabbit or anti-mouse IgG antibodies. Samples were rinsed with PBS 1X, mounted with fluoromount G containing DAPI on glass slides and representative images were acquired in an epifluorescence microscope Nikon *Ti* (Tokio, Japan).

### Silencing of dysferlin by small hairpin RNA

Commercial small hairpin RNA plasmid against dysferlin (Santa Cruz Biotech, Dallas, Tx), with puromycin resistance, was used to transfect RCMH myoblast through lypofectamine method [32]. The silencing of dysferlin was confirmed by western blot analysis.

### Selection of dysferlinopathy patients, mutations and biopsy studies

Following ethical guidelines, healthy volunteers and dysferlinopathy patients who participated in this study signed an informed consent approved by the local Ethics Committee in accordance with the ethical standards laid down in the 1964 Declaration of Helsinki and its later amendments. Diagnosis of dysferlinopathy was achieved based on clinical examination, muscular biopsy and mutation analysis. The mutations of the patients are published in Woudt et al. [33].

### Statistical analysis

Results are presented as mean ± standard error (SE). For multiple comparisons with a single control, a non-parametric one-way ANOVA followed by the Dunn’s multiple comparison test was used. Analyses were carried out using GRAPHPAD software. *P* < 0.05 was considered statistically significant.

## Additional file

Additional file 1: Figure S1. TRPV2 channels are functionally expressed in absence of dysferlin. Human control myotubes, RCMH myotubes (◯); human myotubes that lack dysferlin, small hairpin RNA Dysferlin (□⃞); and myotubes 379 (∇), were loaded with Fura-2 AM dye and the Ca^2+^ elevation, induced by 2-Aminoethoxydiphenyl borate (2-APB), was evaluated and compared with that of RCMH myotubes that express dysferlin. The data represents mean ± SEM. *n* = 4 cell cultures for each cell line. (JPG 371 kb)
