# Supplementary figures and images for: The absence of dysferlin induces the expression of functional connexin-based hemichannels in human myotubes
Source: BMC Cell Biol. 2016 May 24;17(Suppl 1):15. doi: 10.1186/s12860-016-0096-6 (PMC4896263; doi:10.1186/s12860-016-0096-6)

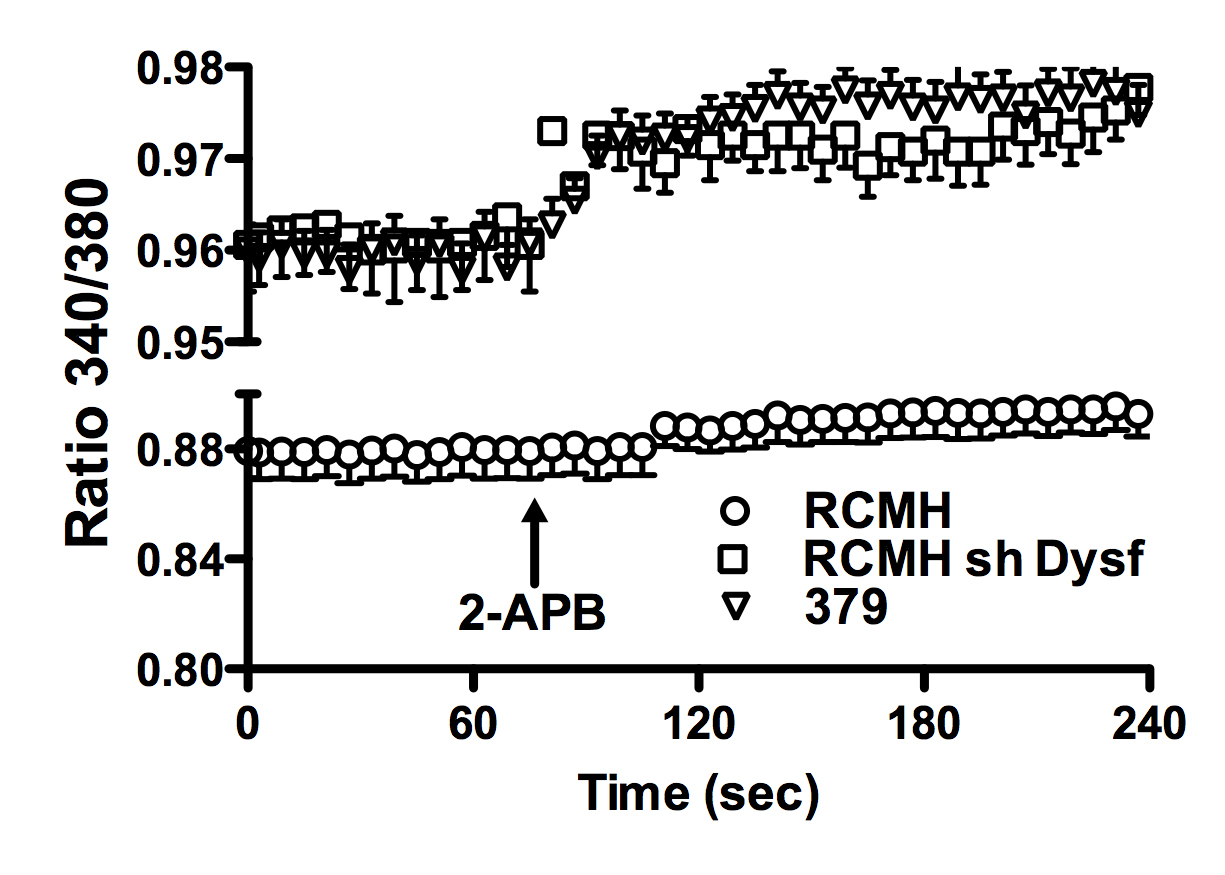

Supplement: Additional file 1: Figure S1. — TRPV2 channels are functionally expressed in absence of dysferlin. Human control myotubes, RCMH myotubes (◯); human myotubes that lack dysferlin, small hairpin RNA Dysferlin (□⃞); and myotubes 379 (∇), were loaded with Fura-2 AM dye and the Ca2+ elevation, induced by 2-Aminoethoxydiphenyl borate (2-APB), was evaluated and compared with that of RCMH myotubes that express dysferlin. The data represents mean ± SEM. n = 4 cell cultures for each cell line. (JPG 371 kb) [file 12860_2016_96_MOESM1_ESM.jpg]
